# Switching from entecavir to tenofovir alafenamide for maintaining complete virological response in chronic hepatitis B

**DOI:** 10.1002/jgh3.12950

**Published:** 2023-07-21

**Authors:** Shun Ishido, Nobuharu Tamaki, Naoki Uchihara, Keito Suzuki, Yuki Tanaka, Haruka Miyamoto, Michiko Yamada, Hiroaki Matsumoto, Tsubasa Nobusawa, Taisei Keitoku, Kenta Takaura, Shohei Tanaka, Chiaki Maeyashiki, Yutaka Yasui, Yuka Takahashi, Kaoru Tsuchiya, Hiroyuki Nakanishi, Jun Itakura, Masayuki Kurosaki, Namiki Izumi

**Affiliations:** ^1^ Department of Gastroenterology and Hepatology Musashino Red Cross Hospital Tokyo Japan

**Keywords:** chronic hepatitis B, complete virological response, entecavir, low level viremia, tenofovir alafenamide

## Abstract

**Background and Aim:**

Hepatocellular carcinoma development can be decreased by achieving and maintaining complete virological response (CVR) in chronic hepatitis B. However, it is unclear whether switching from entecavir (ETV) to tenofovir alafenamide (TAF) could achieve and maintain CVR in patients with low‐level viremia (LLV; HBV DNA ≤ 3.3 log IU/mL) or occasional detectable HBV DNA during ETV treatment. Therefore, we aimed to examine whether the switching from ETV to TAF is effective in achieving CVR in patients with LLV or occasional detectable HBV DNA.

**Methods:**

This study comprised 45 patients who switched from ETV to TAF. All patients received ETV and TAF for >2 years, and the HBV DNA levels were measured every 3 months. Maintaining undetectable HBV DNA during 2‐year period is defined as CVR. The primary endpoint is the CVR rate during ETV and TAF treatment.

**Results:**

The CVR rate for each of the 2 years of ETV and TAF therapy was 33.3% (15/45) and 68.9% (31/45, *P* < 0.01), respectively, and the CVR rate increased by switching from ETV to TAF. In patients with occasional detectable HBV DNA during ETV treatment (22 patients), 15 achieved CVR and 7 maintained occasional detectable HBV DNA. In patients with LLV during ETV treatment (eight patients), three achieved CVR and five had occasional detectable HBV DNA.

**Conclusion:**

Switching from ETV to TAF increases the CVR rate in patients with LLV or occasional detectable HBV DNA and could be an alternative treatment option.

## Graphical abstract

Switching from entecavir to tenofovir alafenamide increases the complete virological response rate in patients with low level viremia or occasional detectable hepatitis B virus DNA and could be an alternative treatment option.

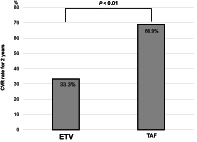

## Introduction

Liver cancer has become the second most common cause of cancer‐related death in males and the sixth in females in 2020.^1^, ^2^, ^3^ Hepatitis B virus (HBV) infection is an important risk factor for hepatocellular carcinoma (HCC) and liver failure.^4^, ^5^, ^6^, ^7^, ^8^ Nucleic acid analogs (NAs) such as entecavir (ETV) and tenofovir alafenamide (TAF) are used for the treatment of chronic hepatitis B. Treatment with NAs reduces HBV DNA levels and could reduce the risk of HCC development.^9^ However, some patients may not achieve negative HBV DNA despite receiving treatment with NAs. Low‐level viremia (LLV), defined as detectable HBV DNA with ≤3.3 log IU/mL (undetectable < HBV DNA ≤ 3.3 log IU/mL) during NA treatment, is a risk factor for HCC development.^10^ Previous research has indicated that patients with complete virological response (CVR), defined as the maintenance of undetectable HBV DNA during treatment, have a low risk of HCC compared to those with occasional detectable HBV DNA.^11^ However, some patients are unable to achieve undetectable HBV DNA and remain with LLV during ETV treatment. Furthermore, although some patients achieve undetectable HBV DNA by ETV treatment, occasional detectable HBV DNA occurs in some other patients.^12^ Recent studies have shown that switching from ETV to TAF helps in achieving undetectable HBV DNA in patients with LLV at a certain point after switching.^13^, ^14^, ^15^ However, it is still unclear whether switching from ETV to TAF could achieve and maintain CVR during treatment with TAF in patients with LLV or occasional detectable HBV DNA. In this study, we aimed to investigate whether the switching from ETV to TAF is effective in achieving CVR in patients with LLV or occasional detectable HBV DNA.

## Methods

### Study protocol

**Figure 1:**
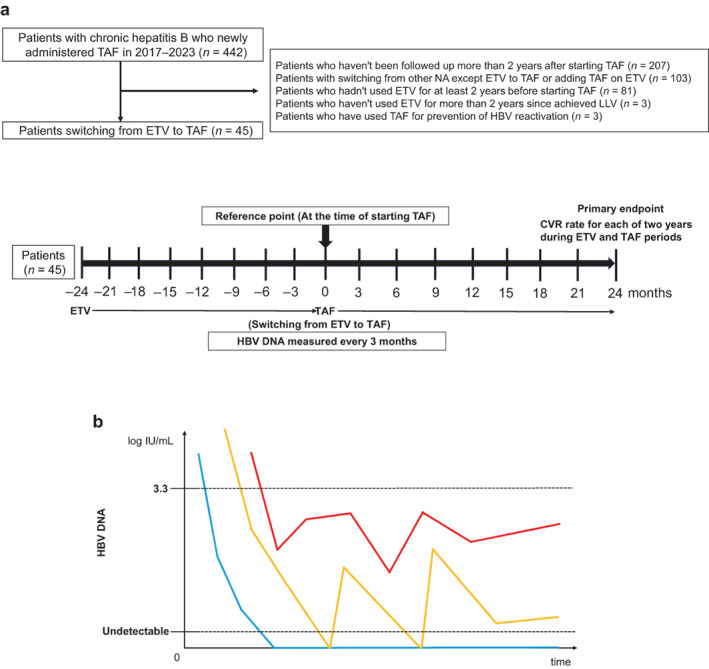
(a) Study flowchart. (b) Conceptual diagram of the definition of hepatitis B virus (HBV) DNA level status, (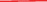), low‐level viremia (LLV); (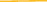), occasional detectable HBV DNA; (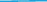), complete virological response (CVR). ETV, entecavir; NA, nucleic acid analog; TAF, tenofovir alafenamide.

This was a single‐center, retrospective study including 442 patients who were newly administered TAF at the Musashino Red Cross Hospital from January 2017 to March 2023. A total of 45 patients with chronic hepatitis B who switched from ETV to TAF were registered, whereas 207 without follow‐up for more than 2 years after starting TAF, 103 who switched from other NAs except for ETV or adding TAF on ETV, 81 who were not using ETV for at least 3 years, 3 who were not using ETV for more than 2 years since achieving LLV, and 3 who were using TAF for the prevention of HBV reactivation were excluded. The study reference point was the time of starting TAF. All patients achieved LLV with ETV treatment and received ETV for more than 2 years after achieving LLV. All patients switched from ETV to TAF and received TAF for more than 2 years. HBV DNA levels were measured every 3 months and the CVR rate was measured. There were 720 opportunities in total to measure HBV DNA in 4 years (16 points for each patient), of which HBV DNA was measured in 711 (98.8%) opportunities. The study flowchart is shown in Figure 1a.

### Clinical and laboratory data

The day TAF was started was used as reference point, and HBV DNA levels were measured every 3 months during 2 years before (during ETV) and after the reference point (during TAF). Quantitative measurement of HBV DNA was done using real‐time PCR (COBAS 6800/8800 system, TaqMan HBV assay; Roche). The serum HBV DNA was measured by real‐time PCR with a detection range of 1.0–9.0 log IU/mL. The lower limit was 10 IU/mL.

### Definition of HBV DNA levels status

HBV DNA remaining undetectable during the 2‐year period is defined as CVR. If patients achieved undetectable HBV DNA once but detectable HBV DNA occasionally, it is defined as occasional detectable HBV DNA. LLV is defined as detectable HBV DNA with ≤3.3 log IU/mL (not achieving undetectable HBV DNA). The conceptual diagram of the definition of HBV DNA status is shown in Figure 1b.

### Primary endpoint

The primary endpoint was the CVR rate for each of the 2 years during ETV and TAF treatment.

### Statistical analysis

McNemar's test was used to compare the CVR rate for each of 2 years during ETV and TAF treatment. *P*‐values of <0.05 were considered statistically significant. All statistical analyses were conducted using EZR.^16^

This study was performed following the principles of the Declaration of Helsinki and with the consent of the ethics committee of the institution where the study was carried out (Approval Number: 29085).

## Results

### Patient characteristics

Patient characteristics on the day of starting TAF are given in Table 1. A total of 45 patients who switched from ETV to TAF were enrolled in the study. The median (interquartile range [IQR]) of the patients' age was 59 (50–69) years. Of these, 23 (51.1%) were males. The median (IQR) HBV DNA levels were undetectable (undetectable, 1.0 log IU/mL). Aspartate aminotransferase (AST) and alanine aminotransferase (ALT) levels were in the normal range (AST, 23 [20–27] IU/L; ALT, 20 [15–21] IU/L). Previous ETV treatment was for 72 (58–108) months. During ETV treatment, LLV, occasional detectable HBV DNA, and CVR were observed in 8, 22, and 15 patients, respectively.

**Table 1:** Patient characteristics on the day of starting tenofovir alafenamide

	Patients (*n* = 45)
Age (years)	59 (50–69)
Sex, male/female	23/22
HBsAg (IU/mL)	925 (110–2103)
HBV DNA (log IU/mL)	Undetectable (undetectable, 1.0)
HBeAg positive/negative	6/39
HBeAb positive/negative	43/2
AST (IU/L)	23 (20, 27)
ALT (IU/L)	20 (15, 21)
Platelet counts (10^9^/L)	179 (158, 207)
Previous ETV treatment (months)	72 (58, 108)
LLV/occasional detectable HBV DNA/CVR during ETV treatment	8/22/15

Data are shown in median and interquartile range.

ALT, alanine aminotransferase; AST, aspartate aminotransferase; CVR, complete virological response; ETV, entecavir; HBeAb, hepatitis B e‐antibody; HBeAg, hepatitis B e‐antigen; HBsAg, hepatitis B s‐antigen; HBV, hepatitis B virus; LLV, low‐level viremia.

### CVR rate for each of the 2 years during ETV and TAF treatment

**Figure 2:**
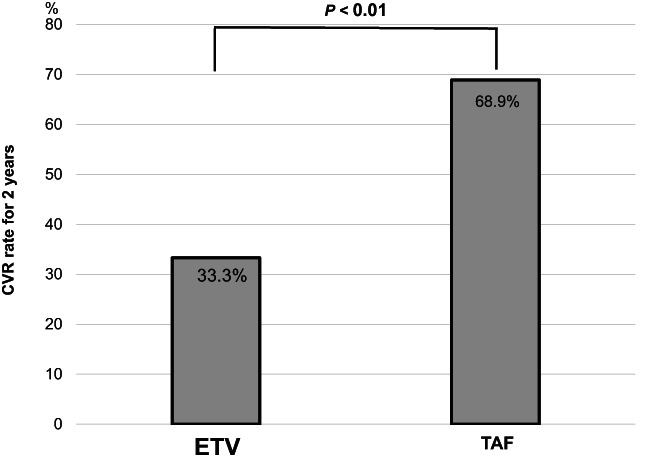
Comparison of complete virological response (CVR) rate for each of the 2 years during entecavir (ETV) and tenofovir alafenamide (TAF) treatment.

HBV DNA was monitored every 3 months; thus these were measured eight times during each of the 2 years of ETV and TAF treatment, respectively. The CVR rate was calculated as the percentage of patients whose HBV DNA was negative at all eight times in each of the 2 years. The number of patients with CVR was 15 (out of 45) during the ETV treatment period and 31 during TAF. The CVR rate for each of the 2 years during the ETV and TAF treatment periods was 33.3 and 68.9% (*P* < 0.01), respectively, and the CVR rate increased by switching from ETV to TAF (Fig. 2).

### Change of HBV DNA level status

**Figure 3:**
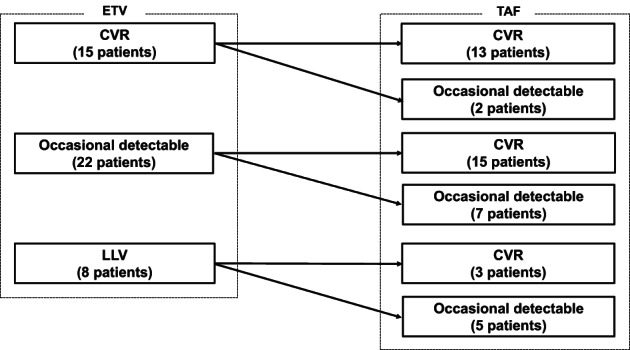
Change of hepatitis B virus (HBV) DNA level status. CVR, complete virological response; ETV, entecavir; LLV, low‐level viremia; TAF, tenofovir alafenamide.

The HBV DNA level status of patients changed as follows: In patients with CVR during ETV (15 patients), 13 patients attained CVR and two patients had occasional detectable HBV DNA. In patients with occasional detectable HBV DNA during ETV (22 patients), 15 patients achieved CVR and 7 patients remained with occasional detectable HBV DNA, and no patients deteriorated to LLV during the TAF treatment period. In patients with LLV during ETV (eight patients), three and five patients achieved CVR and occasional detectable HBV DNA. In patients with occasional detectable HBV DNA and LLV during ETV, 18 patients (60%) had achieved CVR, and the rate increased significantly after switching to TAF (*P* < 0.01). The change in HBV DNA level status is shown in Figure 3.

## Discussion

### Main findings

In this study, we found that the CVR rate increased by switching from ETV to TAF. A previous study had shown that CVR during treatment with NAs could reduce the risk of HCC.^17^ Therefore, switching from ETV to TAF in patients who did not achieve CVR during ETV treatment could be an effective treatment option.

### Comparison with published literature

HBV DNA was detectable in about 20% of patients during treatment with NAs.^13^ Host characteristics such as age and sex were found to be associated with HCC development.^18^, ^19^, ^20^ Regarding the viral factor for HCC development, LLV is a known risk factor for HCC development,^10^ and avoiding LLV reduces the risk of HCC development in patients with chronic hepatitis B.^10^, ^11^, ^21^, ^22^ Previous studies have shown the utility of switching from ETV to TAF.^23^, ^24^, ^25^ Regarding HBV DNA decline, switching from ETV to TAF in patients with LLV could increase undetectable HBV DNA rate at a point after switching (24, 48, or 144 weeks).^13^, ^14^, ^15^ However, there have been only limited data on whether switching from ETV to TAF could maintain CVR.^26^ In this study, we found that the CVR rate increased after switching from ETV to TAF. According to the Japan Society of Hepatology guidelines for HBV treatment,^27^ the therapeutic goal of treatment with NAs is the achievement of undetectable HBV DNA. Therefore, switching from ETV to TAF can be an optimal treatment option in patients with LLV or occasional detectable HBV DNA.

In this study, we had switched from ETV to TAF in patients with CVR. The main reason for switching from ETV to TAF was to improve medication satisfaction, adherence, or to reduce HBV surface antigen.^25^, ^28^ However, the clinical significance of switching from ETV to TAF in patients with CVR during ETV needs further studies.

### Strengths and limitations

The strength of this study is that almost all patients received blood tests every 3 months, which allowed for accurate measurement of the amount of HBV DNA and sustained negativity for 2 years. As for the limitation of this study, it was performed at a single institution (in Japan only) and there were a limited number of cases. Therefore, further multicenter studies are needed to confirm the effect of switching from ETV to TAF. In addition, results were available only for 2 years, so further studies over long periods are required and are needed to compare CVR in patients who continued with ETV and those who switched from ETV to TAF.

### Future implications

In this study, we demonstrated that switching from ETV to TAF could be an alternative treatment option in patients with LLV or occasional detectable HBV DNA. CVR is associated with a lower risk of HCC development in patients with chronic hepatitis and cirrhosis.^10^, ^11^ Although a further longitudinal study is required, maintaining CVR by switching from ETV to TAF may reduce HCC development in these patients. Recent studies have shown that tenofovir is superior to ETV in suppressing HCC development.^29^ One aspect of the result may be associated with medication adherence. Poor medication adherence is associated with a high HCC development rate.^30^ Switching from ETV to TAF helps to improve medication adherence.^28^ Although medication adherence is not examined in this study, improving medication adherence may be associated with maintaining CVR and may also contribute to improving HCC development. A larger longitudinal study on whether switching from ETV to TAF can reduce HCC development is required. In conclusion, switching from ETV to TAF increases the CVR rate in patients with LLV or occasional detectable HBV DNA and could be an alternative treatment option.

## Patient consent

We collected informed consent from all patients using an opt‐out approach.

## Data Availability

The datasets generated and analyzed during the current study are not publicly available to protect personal information, but they are available from the corresponding author upon reasonable request.
